# Determination of Fracture Mechanism and Mode II Fracture Toughness of Red Sandstone Subjected to Compressive-Shear Loading

**DOI:** 10.3390/ma19061236

**Published:** 2026-03-20

**Authors:** Chang-Hong Lei, Huai-Zhong Liu, Hong-Qiang Xie, Ming-Li Xiao, Gan Feng, Zhao-Qiang Zheng

**Affiliations:** State Key Laboratory of Hydraulics and Mountain River Engineering, College of Water Resource and Hydropower, Sichuan University, Chengdu 610065, China; leich1@stu.scu.edu.cn (C.-H.L.); alex_xhq@scu.edu.cn (H.-Q.X.); xiaomingli@scu.edu.cn (M.-L.X.); fenggan@scu.edu.cn (G.F.);

**Keywords:** mode II fracture toughness, compressive shear, shear box test, digital image correlation, crack initiation angle

## Abstract

Mode II fracture toughness is an important material parameter of rocks, but accurate measurement of this parameter is still a challenge in rock fracture mechanics. This study aims to modify the mode II fracture toughness of red sandstone measured through shear box testing by emphasizing the critical role of crack initiation angle. Experimental tests combining fracture trajectory scanning and digital image correlation reveal distinct fracture mechanisms of red sandstone under varying loading angles: tensile spalling dominates low angles, and shear fractures emerge at medium angles, while tensile fracture initiates from the rock bridge center at high angles. Although shear fracture initiates from the notch tip, its initiation angle deviates from the initial crack plane, invalidating traditional mode II fracture toughness determination methods. A modified Mohr–Coulomb criterion incorporating fracture angle and Mode I stress intensity factor is proposed to correct the significant errors of traditional methods, and this study establishes a refined framework for mode II fracture toughness determination under compression–shear conditions.

## 1. Introduction

Rock fracture mechanics is one of the core theoretical foundations for deep resource development, stability assessment of underground engineering, and prevention and control of geologic hazards [1,2,3,4]. Engineering rock mass can become instable under the impact of engineering disturbance, such as shear slip induced by water injection and rockbursts caused by fault activation [5]. In such cases, shear fracture serves as one of the core mechanical drivers of rock failure. Thus, it is necessary to find suitable methods and configurations to test mode II fracture toughness. According to the loading conditions, the existing mode II fracture testing methods can be categorized into two types—pure mode II loading and compressive-shear loading—and the difference in mechanical responses between the two significantly affect the fracture toughness test results.

In terms of pure shear loading, researchers have mainly focused on the continuous optimization of specimen geometry and loading method to achieve pure mode II fracture. Brazilian disc type specimens (e.g., CBD specimen [6] and DCFBD specimen [7]) are widely used in mode II fracture studies because of convenience for loading and theoretical solving. Ayatollahi and Sistaninia [6] carried out mode II fracture tests in different rocks using a centrally cracked Brazilian disc (CBD), and the analysis showed that the T-stress at the tip of the crack (a non-singular stress term) affected the mode II fracture toughness test results; based on this finding, Ayatollahi and Aliha [8] improved the maximum tangential stress criterion (MTS) and proposed a mode II fracture criterion with T-stress correction. However, Ji et al. [9] and Lin et al. [10] conducted full-field strain monitoring of Brazilian disc and semicircular bending (SCB) specimens using a digital image correlation (DIC) technique and found that the dominant mechanism of crack propagation in the absence of confining pressure was mode I fracture. Consequently, they held that a stable mode II fracture path could only occur with the existence of confining pressure. The above studies reveal the inherent contradiction of pure shear loading: although geometric design and loading optimization can approximate the ideal shear stress field, crack extension is inevitably accompanied by tensile stress, which ultimately leads to mode I fracture or mixed-mode fracture [10,11]. In order to avoid the transition of fracture mechanism from mode II to mode I fracture, reducing the rock bridge length of shear fracture or increasing the initial crack length can promote the stable propagation of mode II fracture. For example, Cao et al. [12] proposed the Z-shaped center-cracked straight shear (ZCCDS) method to test the mode II fracture toughness of rocks, and it was found that with an increase in the initial crack length, the fracture mechanism gradually changed from I/II mixed-mode fracture to pure mode II fracture. Watkins and Liu [13] developed the Short Beam Compression (SBC) method and found that shear fracture of rock bridges can be observed when the rock bridge length is between 0.1 and 0.25 times of the specimen length. Xu et al. [14] further improved SBC and proposed the short core in compression (SCC) method. Their tests on Fangshan marble revealed that the fracture mode of the rock bridge gradually transitioned from tension fracture to mode II fracture as the length of the rock bridge shortened, and the results of the mode II fracture toughness test were basically consistent with that produced by the ISRM-suggested punch-through shear (PTS) method.

Applying compression–shear loading is another solution for inhibiting tension crack extension and promoting mode II fracture. Research on mode II fracture under compression–shear loading conditions has also made remarkable progress in recent years. In 2012, the punch-through shear (PTS) test designed by Backers and Stephansson [15] was suggested by the ISRM as a test standard for mode II fracture toughness. This test is performed by applying triaxial stress to the specimen containing annular slit by means of a circumferential pressure chamber so that the specimen undergoes shear fracture under the action of the circumferential pressure. Additionally, by changing the angle of the initial crack of disc and half-disc fracture specimens, the initial crack can also be put under compression–shear loading. Chen et al. [16] applied compression–shear loading to the crack using the double-notched Brazilian disc (DNBD) method, and the normal stress on the crack inhibited the deflection of the tensile cracks and caused the fracture to expand along the initial crack plane. The experimentally measured *K*_IIC_ of granite was increased by about 30% compared with that under the unconfined condition, which confirms the reinforcing effect of confining pressure on shear fracture. Rao et al. [17] proposed a shear box fracture test to investigate mode II fracture toughness and suggested the dimensions of specimens. Using the proposed method, they further analyzed the mode II fracture toughness of granite, marble, and sandstone using the Mohr–Coulomb criterion. Based on the shear box test, Sun et al. [18] and Cao et al. [19] studied the mechanical behavior and shear fracture toughness of transversely isotropic rock. Although compressive-shear loading methods have significantly improved the reliability of mode II fracture tests, these compressive-shear test methods still rely on short rock bridges to ensure that the shear fracture propagates along the initial crack plane, whereas under the condition of a longer rock bridge, rock probably produces compressive-shear fracture that deviates from the initial crack plane. For example, Ying et al. [20] carried out compression–shear tests on unilateral notched specimens based on the principle of the shear box test, and they found that the normal pressure not only enhances the *K*_IIC_ but also changes the crack initiation angle, which is greater than 5°.

**Figure 1:**
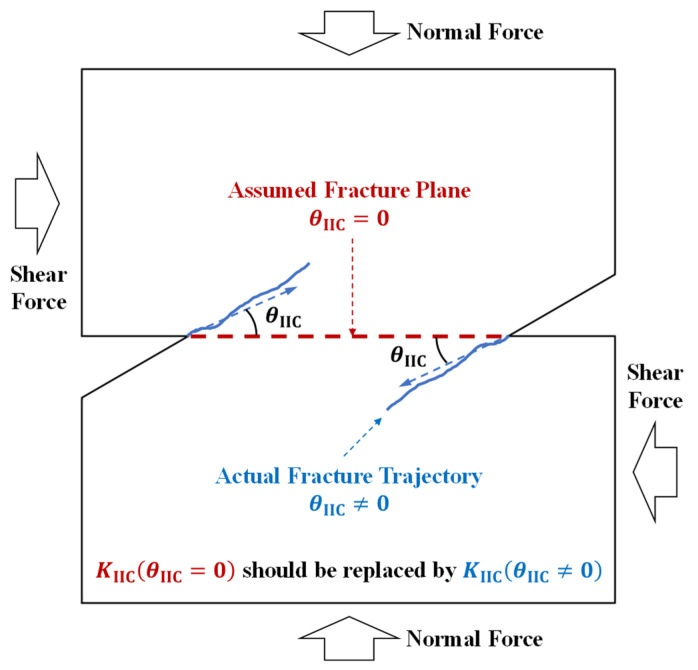
The improvement of the determination of mode II fracture toughness. (The blue arrows indicate the direction of cracking).

In the fracture toughness analysis of the above-mentioned compression–shear test, it was assumed that the fracture initiated along the initial crack plane, and the effect of the crack initiation angle on the mode II fracture toughness was not taken into account. To address this problem, the compression–shear cracks deviating from the initial crack plane in the shear box test are taken as the object of this study, and the solution method for mode II fracture toughness was modified by considering the crack initiation angle, i.e., *K*_IIC_(*θ*_IIC_ = 0) should be replaced with *K*_IIC_(*θ*_IIC_ ≠ 0) to determine the mode II fracture toughness by considering the crack initiation angle *θ*_IIC_, as shown in Figure 1. Firstly, a batch of bilaterally cut square red sandstone specimens with rock bridge lengths greater than the recommended value for the shear box test were produced, and compression–shear fracture tests were carried out using the shear box test; the fracture mechanism of the specimens was analyzed by using the full-field strain measured by the DIC technique. Finally, a compression–shear fracture toughness solution method was established based on the Mohr–Colomb criterion, and the mode II fracture toughness of red sandstone was analyzed considering the crack initiation angle.

## 2. Shear Box Test

### 2.1. Specimen and Experimental Equipment

**Figure 2:**
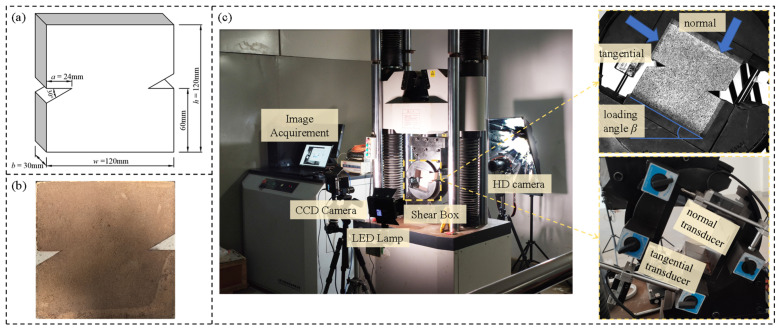
Test specimen and equipment: (**a**) geometric dimensions of specimen, (**b**) rock specimen, and (**c**) shear box test system and DIC monitor device.

The red sandstone used in this study was collected from Sichuan Province, China. According to the results of compression tests conducted on the red sandstone, the elastic modulus of red sandstone is 3.24 GPa, Poisson’s ratio is 0.279, the cohesion is 3.29 MPa, and the friction coefficient is 1.11. The moisture content of the red sandstone specimens is 0.61%, and the total porosity measured by the pycnometer method is 14.72%. Through micro-morphology observation via scanning electron microscopy (SEM), the grain size of the tested red sandstone ranges from 0.07 mm to 0.13 mm. Dense cement fills the gaps between the clastic particles, and the particles are tightly bonded with the cement. Almost no visible pre-existing microcracks are observed. The sandstone exhibits excellent homogeneity and can be regarded as an isotropic material. Rock blocks were processed into square plates of 120 mm × 120 mm × 30 mm, and a triangular notch with a depth of *a* = 24 mm was cut out from the center of the two side edges as the initial notch (Figure 2). The advantage of the triangular notch is that the problem of notch closure does not occur in the process of continuous pressurization, which can effectively avoid the impact of closed cracks on the test. The relative crack depth 2*a*/*W* = 0.4, which is less than the 0.6 suggested by Rao et al. [17], was set to study the compression–shear fracture initiating away from the initial crack plane.

Shear box tests were carried out on a servo-hydraulic testing machine, where a steel shear box was placed on the testing machine, which allowed the specimen be loaded at different loading angles to produce adjustable stress ratios, as shown in Figure 2c. Five test groups were designed, with the loading angle *β* set at 30°, 40°, 50°, 60°, and 70°, respectively. Liu et al. [21] recommended keeping the loading angle β below 70° in shear box tests because the specimen will not keep sufficient contact with the loading device when the loading angle exceeds 70°. Thus, the maximum loading angle is determined to be 70°. To better suppress tensile cracks and promote compressive-shear failure, the loading angle was reduced to increase the normal force in the shear box test. Therefore, a series of angles smaller than 70° was selected, with the minimum loading angle set at 30°. To maximize the range of loading angles tested within a limited total number of specimens, two specimens per group were assigned, and the configuration could not be expanded due to the inability to obtain additional specimens with comparable properties.

The geometrical dimensions, masses, and loading angles of the specimens are listed in Table 1. All tests were conducted using the same equipment and under identical laboratory conditions, so the primary source of measurement uncertainty originates from the test specimens themselves. Regarding the specimens, rock heterogeneity and specimen machining dimensions could influence the test results. First, rock heterogeneity can be reflected by its density. The density of these specimens ranges from 2.093 to 2.197 g/cm^3^, with a coefficient of variation (CV) of 1.33%. Second, the specimen dimensions also exhibit limited variability: side length ranges from 119.6 to 120.33 mm (CV = 0.14%), thickness from 30.11 to 30.39 mm (CV = 0.33%), and notch depth from 23.63 to 24.26 mm (CV = 0.64%). These variations introduce slight differences in geometry and material properties, which, in turn, contribute to the scatter in the measured mode II fracture toughness across specimens.

Since a high confining pressure could suppress the initiation of tensile fracture, loading angles of 30° and 40° were set to achieve a high-normal pressure on the specimen. In the test, the external load was applied in the vertical direction of the machine at a fixed axial displacement rate of 1 mm/min. Throughout the loading process, the axial load was recorded by a load transducer, and the tangential and normal displacements of specimens were recorded by displacement transducers placed in the tangential and normal directions, where tangential and normal directions are along and perpendicular to the plane connecting two notch tips, respectively.

To better determine the fracture mechanism of the specimen, the DIC technique was applied to obtain images for analysis [22]. The DIC measurement system consists of a high-speed camera, two LED lamps, and a computer to collect images. The camera is an industrial digital camera from Daheng Imaging (Beijing, China), with pixels of 2448 × 2048. The high-speed camera was placed perpendicular to the spotted free surface of the specimen at a proper distance to ensure a clear and complete view of the specimen throughout loading. LED lamps were fixed on both sides of the camera to provide uniform and soft illumination. During testing, the real-time image of the rock was recorded at a rate of 1 image/s. At the same time, an ordinary color digital camera was arranged behind the specimen to acquire high-definition images of the rear surface of the specimen at a frequency of 1 image/s. Compared with the front surface sprayed with scattered spots, the image of the rear surface can visualize the generation and expansion of cracks on the surface of the specimen as well as the fracture pattern of the specimen.

### 2.2. Load-Displacement Curve

**Figure 3:**
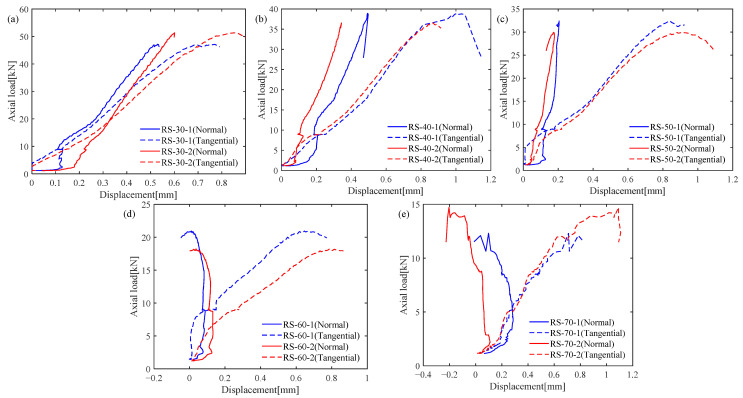
Load-displacement curves of red sandstone under loading angles of (**a**) 30°, (**b**) 40°, (**c**) 50°, (**d**) 60°, and (**e**) 70°.

The variations of axial load, axial displacement, normal displacement, and tangential displacement were measured during the shear box tests. Here, the axial load–tangential displacement curve and axial load–normal displacement curve are used to observe the deformation and load characteristics of the specimens under different loading angle conditions. As shown in Figure 3, the tangential displacements of all the specimens keep increasing during the loading process, and the maximum axial load decreases as the loading angle increases. But the variations of normal displacements are significantly affected by the loading angle and present two different types. The first type is that the normal displacement keeps increasing during the loading process, for example, under loading angles of 30°, 40°, and 50°. The normal deformation mechanism of the first type is dominated by compression. The second type is that the normal displacement increases initially and then decreases to a negative value, for example, under loading angles of 60° and 70°, so the normal deformation mechanism of the second type is finally dominated by shear dilation.

**Figure 4:**
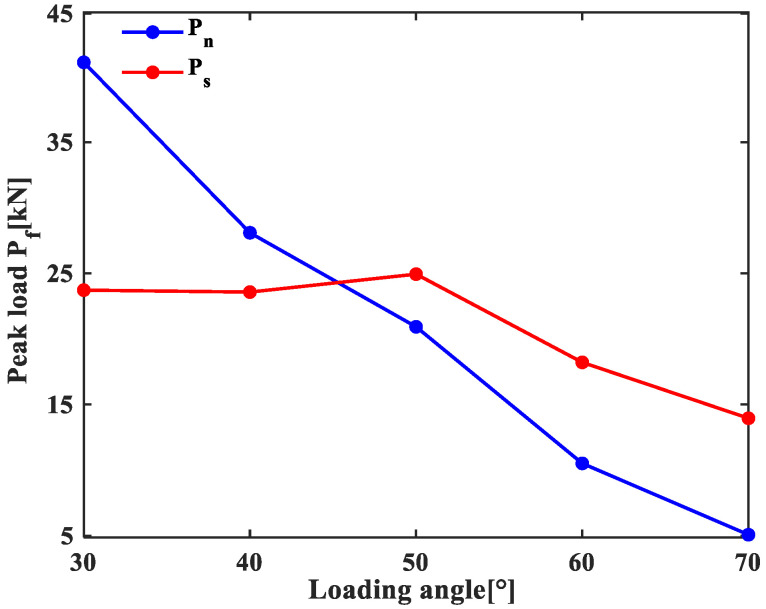
Variation of average normal and tangential forces with the loading angle.

The normal force and tangential force on the specimen are $P_{n}=P\cos\beta$ and $P_{s}=P\sin\beta$, respectively. Under the action of peak load, the average normal forces and tangential forces on the specimens are shown in Figure 4. With the increase in loading angle, the normal force decreases significantly, whereas the tangential force undergoes a gentle decrease.

### 2.3. Fracture Characteristics

**Figure 5:**
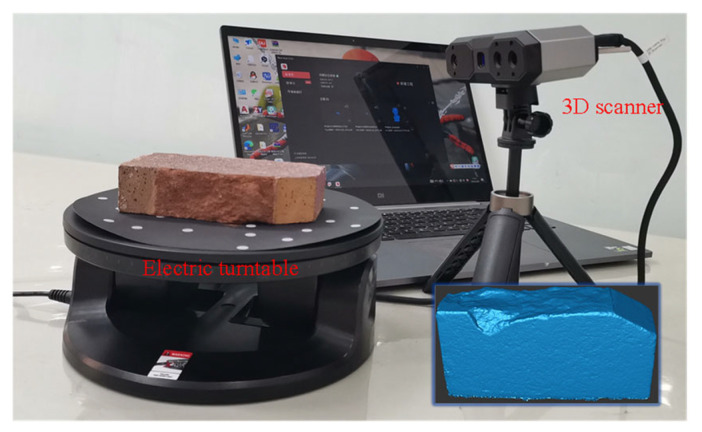
Fracture trajectory scanning with a 3D scanning device.

**Figure 6:**
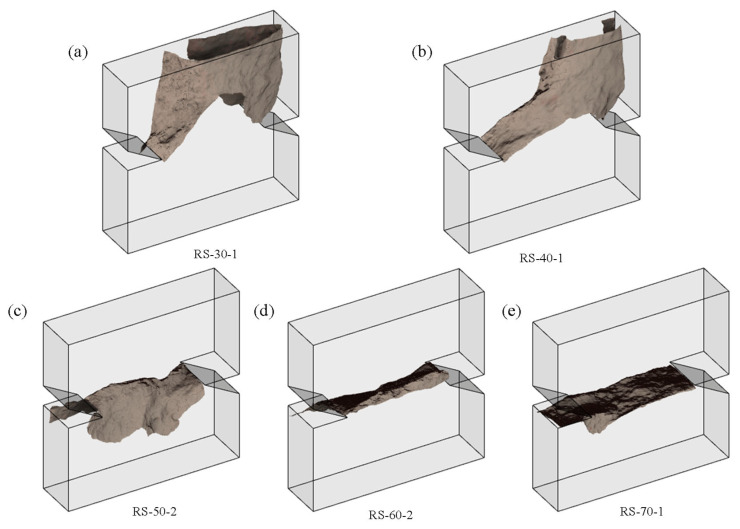
3D scanning images of the fracture trajectory of specimens under loading angles of (**a**) 30°, (**b**) 40°, (**c**) 50°, (**d**) 60°, and (**e**) 70°.

To better study the fracture pattern, a 3D scanner was used to scan the fracture trajectory, and the scanning setup is shown in Figure 5. According to the 3D fracture trajectory scanning results, the fracture of specimens can be classified into two patterns, i.e., chaotic fracture and regular fracture. As shown in Figure 6a,b, specimens loaded under loading angles of 30° or 40° exhibit a chaotic fracture pattern. The spalling fracture grows towards the specimen’s free surface, and the fracture trajectories on the front and back surfaces are completely different. Although a high-normal pressure can suppress the initiation of tensile fracture, it brings another problem in the shear box fracture test. The normal force of the specimen under a load of 30° or 40° is greater than the shear forces, so the normal force dominates the fracture of specimens. These maximum normal forces range from 28.1 kN to 44.6 kN and are equivalent to a boundary stress of 7.8 MPa to 12.4 MPa along the normal direction. Under such high compressive stresses, red sandstone specimens would fail in a tensile spalling way, so their fracture trajectories are chaotic. For this reason, it is not recommended to conduct shear box fracture tests under a loading angle below 45°.

Specimens under load angles of 50°, 60°, and 70°, however, present a regular fracture pattern. As shown in Figure 6c–e, the fracture trajectories on the front and back surfaces are almost the same. The fracture surface near the notch tip is smooth and shows signs of sliding, but a bumpy phenomenon is observed in the middle part of the specimen. It indicates that the cracks may initiate in a shear fracture mode but then shift to tensile fracture after propagating for a certain distance [23].

**Figure 7:**
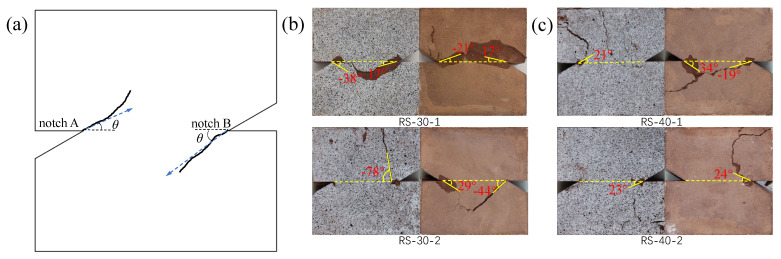

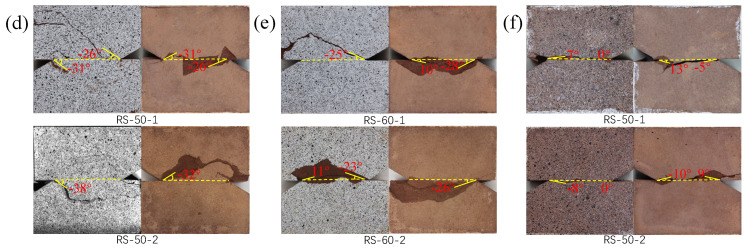
Destruction states of red sandstone specimens: (**a**) positive direction of fracture angle, (**b**) *β* = 30°, (**c**) *β* = 40°, (**d**) *β* = 50°, (**e**) *β* = 60°, and (**f**) *β* = 70°. (The blue arrows indicate the direction of cracking).

According to the custom in fracture mechanics [24], the direction of fracture extension is positive in the counterclockwise direction from the initial notch, as shown in Figure 7a. The final fracture patterns of the specimens are shown in Figure 7, where the fracture angles of the front (scattered surface) and back of the specimen are labeled. The fracture order of two notches and consistency of the front and back fractures were also counted according to the fracture of the specimens [25], as detailed in Table 2.

**Figure 8:**
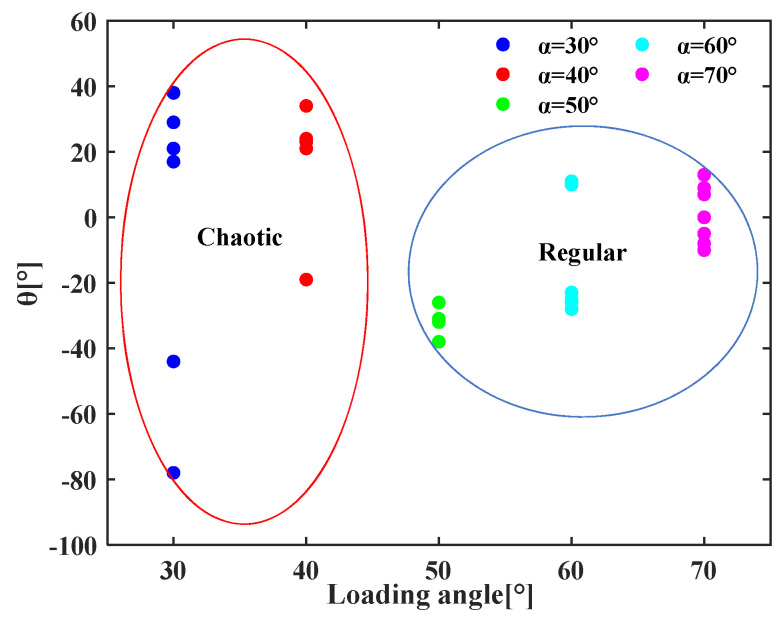
Crack initiation angles under various loading angles.

To better analyze the effect of the loading angle on the crack initiation angle, the data in Table 2 is processed into a scatter plot of crack initiation angle versus loading angle, as shown in Figure 8. It shows that the variation in the crack initiation angle at loading angles of 30° and 40° is remarkable. It indicates that the fracture behavior of the rock is chaotic at these two loading angles, and the direction of crack initiation is also unstable due to the influence of the complex propagation of cracks. With the increase in the loading angle, the crack initiation angle gradually tends to be zero. It implies that when the loading angle ranges from 50° to 70°, the shear stress may dominate the rock fracture. As a result, the fracture mode of the rock tends to be shear fracture, and the cracks are more likely to form a penetrating fracture along the initial crack plane.

## 3. Analysis of Fracture Mechanism by DIC Technique

### 3.1. Deformation Characteristics

It is difficult to directly identify the fracture mechanism of these specimens from the photographs of cracks. Therefore, the DIC technique was used to achieve this goal. Since the failures of the specimens under loads of 30° and 40° are chaotic, only the fracture mechanisms of specimens under the loading angles 50, 60°, and 70° are analyzed in this section. Lin et al. [26] analyzed the displacement state on the fracture surface to identify the fracture mechanism based on the full-field displacement data obtained by the DIC system. When a mode I fracture initiates, normal tensile displacement can be observed between two sides of a crack in the normal direction of the fracture path, whereas relative tangential shear displacement can be detected along the fracture path when a mode II fracture occurs; when I/II mixed-mode fracture occurs, both tensile displacement and shear displacement can be observed.

**Figure 9:**
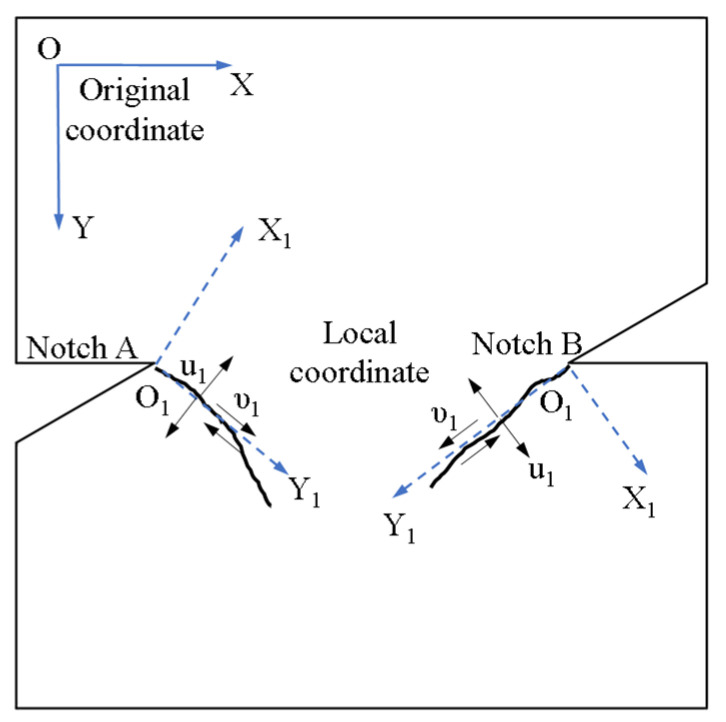
Coordinate systems in the DIC analysis.

The DIC camera (Daheng Imaging, Beijing, China) captures one image per second, followed by DIC analysis using Ncorr (Version 1.2.2 6/13/2017) open-source software. This study selects the region of interest (ROI) as the specimen sizes, with the subset radius set to 20 pixels and subset spacing set to 2 pixels. Ncorr employs dual quintic spline interpolation as its default method for subpixel grayscale value calculation. This method constructs a continuous grayscale surface by fitting quintic polynomials to the grayscale values of 36 surrounding pixels around the target point. Ncorr does not use a fixed correlation coefficient but instead utilizes the Gauss–Newton nonlinear iterative least-squares method to determine the optimal correlation coefficient, establishing a metric for similarity between the final reference and current subsets. During strain field analysis, Green–Lagrangian strain is used for the calculation, and the strain radius is set to 7. In this software, the default coordinate system is the XOY coordinate system, as shown in the upper left corner of Figure 9. Through the secondary development of Ncorr, the XOY coordinate system is converted to the coordinate system X_1_O_1_Y_1_ of the fracture surface (O_1_ is the crack tip, the Y_1_-axis is parallel to the fracture initiation direction, and the X_1_-axis is perpendicular to the initiation direction). After that, the displacement component *u*_1_ can represent the normal displacement perpendicular to the initiating fracture, and the displacement component *v*_1_ can represent the tangential displacement parallel to the initiating fracture.

**Figure 10:**
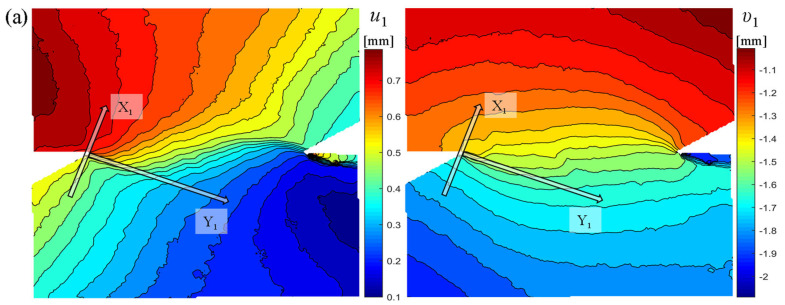

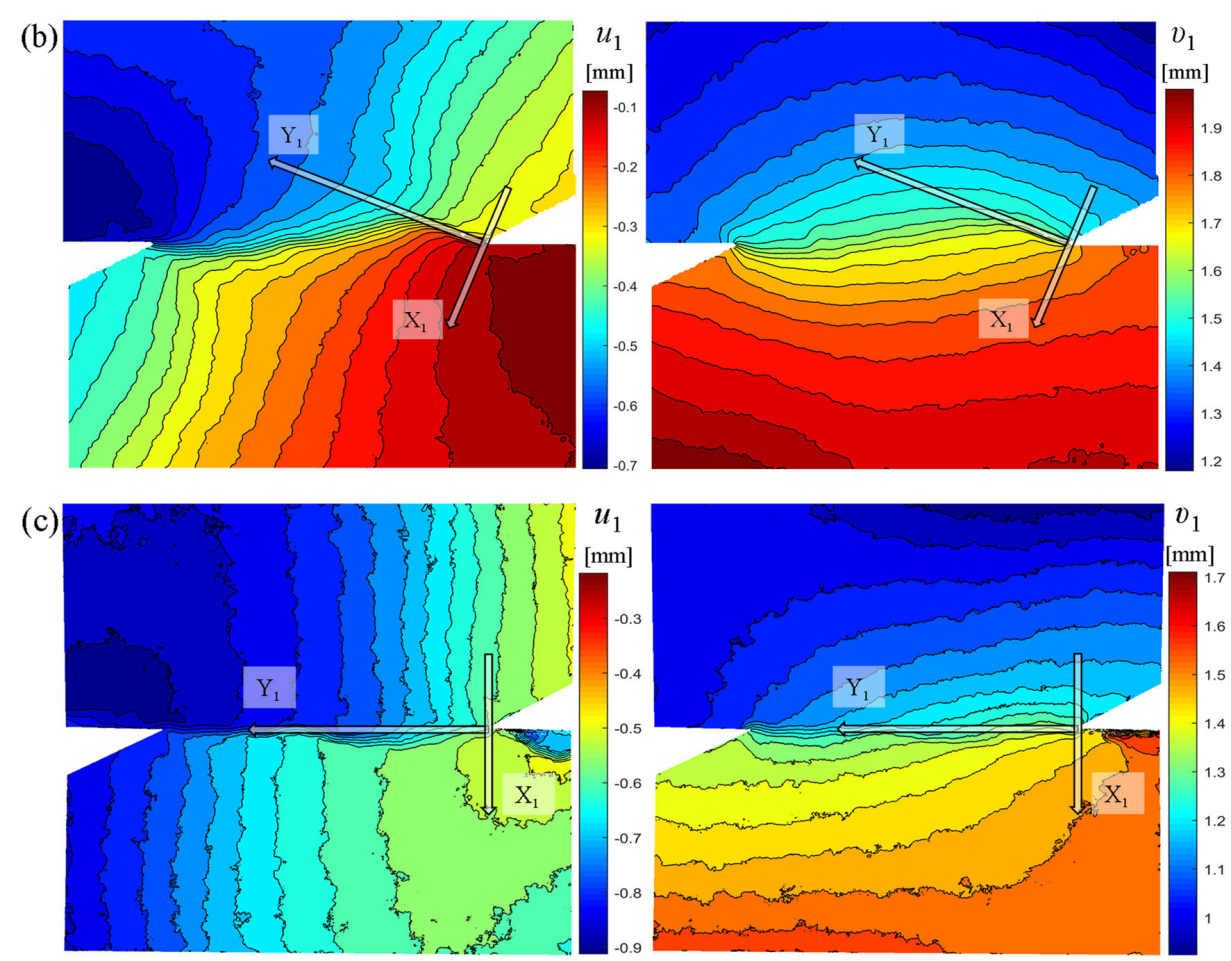
Displacement component of specimens in local coordinate system under peak load: (**a**) RS-50-2, (**b**) RS-60-1, and (**c**) RS-70-1.

Among the specimens with loading angles of 50°, 60°, and 70°, specimens RS-50-2, RS-60-1, and RS-70-1 are selected for illustration, respectively. Figure 10 shows the distribution of displacement components in the local coordinate system for these three specimens under the action of peak load. Under a loading angle of 50°, notch A of RS-50-2 is the first to crack, and a local coordinate system is established in notch A, as shown in Figure 10a. The contours of the displacement component *u*_1_ turns its direction by tens of degrees when passing through the Y_1_-axis, indicating that there is a compressive displacement on the fracture surface. In addition, the contours of displacement component *v*_1_ are almost parallel to the Y_1_-axis and become denser near the Y_1_-axis, indicating that there is a shear displacement on the fracture surface. Thus, it can be preliminarily judged that the fracture mechanism of specimen RS-50-2 is a compression–shear fracture. For specimen RS-60-1, notch B is the first to fracture, and a local coordinate system is established in notch B, as shown in Figure 10b. It shows that the distribution of tangential and normal displacements is similar to those under a loading angle of 50°, which indicates that a loading angle of 60° should also be a compression–shear fracture. The loading angle of RS-70-1 is 70°, and a local coordinate system is established at notch B, as shown in Figure 10c. The contour of displacement component *u*_1_ turns its direction by almost 90 degrees when passing through the Y_1_-axis, and the displacement difference between two sides of the fracture surface is larger than 0.2 mm at the center of the rock bridge, so there is a tensile displacement on the fracture surface. In addition, the displacement component *v*_1_ also develops parallel to the Y_1_-axis and become denser near the Y_1_-axis, indicating that there is a shear displacement on the fracture surface. Thus, the fracture mechanism of specimen RS-70-1 may be a tensile-shear fracture.

### 3.2. Strain Characteristics

**Figure 11:**
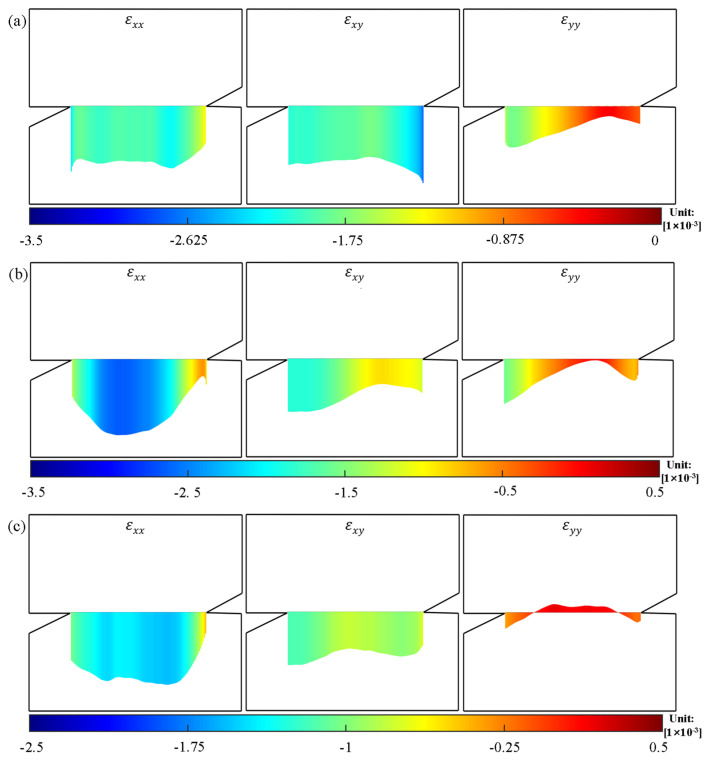
Distribution of strain component on the rock bridge during the elastic phase: (**a**) RS-50-2, (**b**) RS-60-1, and (**c**) RS-70-1. Negative values represent compressive strains.

**Figure 12:**
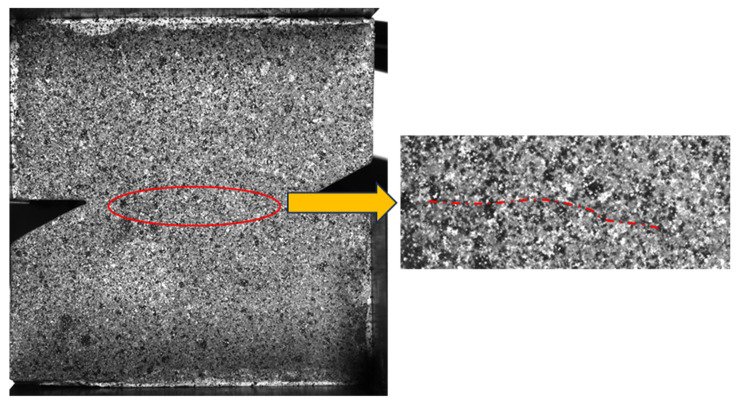
Initial fracture location of RS-70-1. (The red line indicates a crack).

For specimens under a loading angle of 70°, it is not convincible to judge the fracture mechanism by the displacement contour, so it is necessary to analyze the stress state at the tip of the notch and on the rock bridge. In the analysis of strain, the XOY coordinate system was established along the direction of the rock bridge and its normal direction. Figure 11 shows the strain distribution on the notch tip and rock bridge of specimens RS-50-2, RS-60-1, and RS-70-1 in a stress state, where the load is 75% of the peak load before fracture initiation. In the figure, $\varepsilon_{\mathrm{xx}}$, $\varepsilon_{\mathrm{xy}}$, and $\varepsilon_{\mathrm{yy}}$ represent the strains of the rock bridge on the initial crack plane. It shows that before fracture initiation, shear strain $\varepsilon_{\mathrm{xy}}$ can be observed on the notch tips and rock bridges of the three specimens, indicating the presence of shear stress. What is more, compressive strain was observed on the notch tips and rock bridge in the specimens RS-50-2 and RS-60-1, indicating that the notch tips and rock bridge were in a compressive state when the loading angles were 50° and 60°. However, specimen RS-70-1 exhibited tensile strain in the center of the rock bridge, although compressive strain still existed around the crack tip, which disproved the compressive-shear stress state in the middle of the rock bridge. As shown in Figure 12, the high-speed camera captured the phenomenon that cracking first occurred at the center of the rock bridge of RS-70-1. It indicated that when the loading angle was 70°, tensile stress existed in the middle of the rock bridge, and the specimen failed to yield a compression–shear stress state. Resultantly, a tensile fracture initiated from the middle of the rock bridge.

### 3.3. Fracture Mechanism of Crack Tip

**Figure 13:**
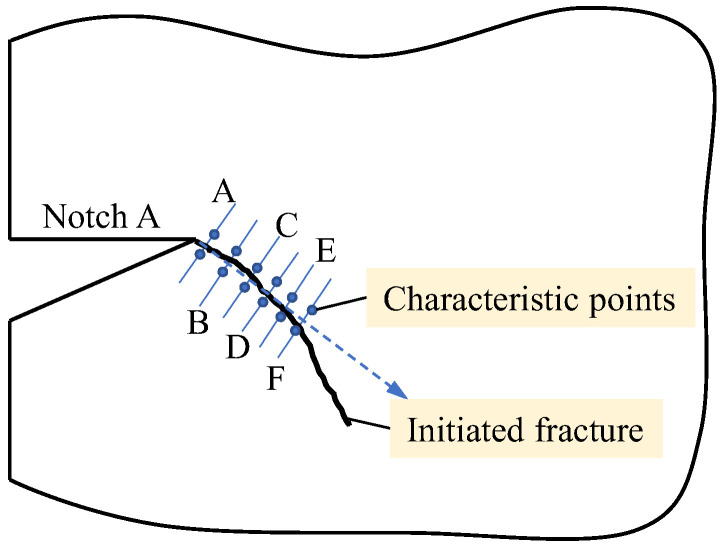
The relative position of the characteristic points to the initiated fracture plane. (The blue arrow indicates the direction of cracking).

For better understanding of the fracture mechanism of red sandstone specimen in the shear box test, six sets of characteristic points were symmetrically selected on both sides of the fracture surface of each specimen (X_1_ = ±0.6 mm). Correspondingly, six profiles were formed perpendicular to the fracture surface, whose positional relationship with the initiated crack plane is shown in Figure 13, and the distances of the six profiles from the notch tip are 1 mm, 2 mm, 3 mm, 4 mm, 5 mm, and 6 mm, respectively. The relative normal and tangential deformation between each two characteristic points is then analyzed to reveal the fracture mechanism of the fracture initiated from the crack tip.

**Figure 14:**
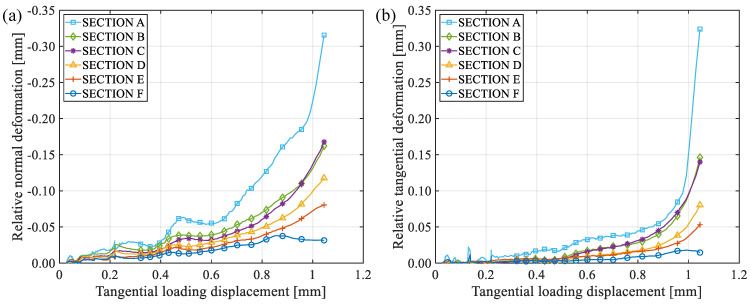
The changes in relative normal and tangential deformations between each two characteristic points of specimen RS-50-2 during the loading process: (**a**) normal deformation; (**b**) tangential deformation.

Figure 14 shows the changes in relative normal and tangential deformations between each two characteristic points of specimen RS-50-2 during the loading process, where the abscissa axes are the tangential loading displacement of the specimen to indicate the loading process. As the test proceeds, the absolute values of relative normal and tangential deformations beside the fracture surface keep increasing until the end of the test. The closer to the notch tip, the greater the relative deformation. The relative normal deformation is negative, indicating compressive deformation. Thus, there are both normal compression displacements and sliding displacements along the fracture surface, and the fracture mechanism is a compressive-shear mixed-mode fracture.

**Figure 15:**
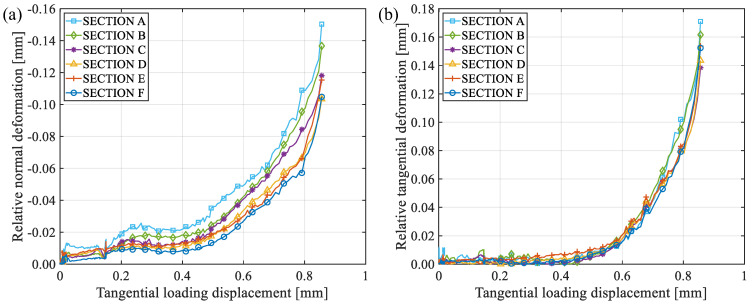
The changes of relative normal and tangential deformations between each two characteristic points of specimen RS-60-1 during the loading process: (**a**) normal deformation; (**b**) tangential deformation.

Figure 15 shows relative normal and tangential deformations between each two characteristic points of specimen RS-60-1 during the loading process. The deformation variations of specimen RS-60-1 are basically the same as those of specimen RS-50-2, so the fracture mechanism is also compressive-shear mixed-mode fracture.

### 3.4. Characteristics of Fracture Surface

**Figure 16:**
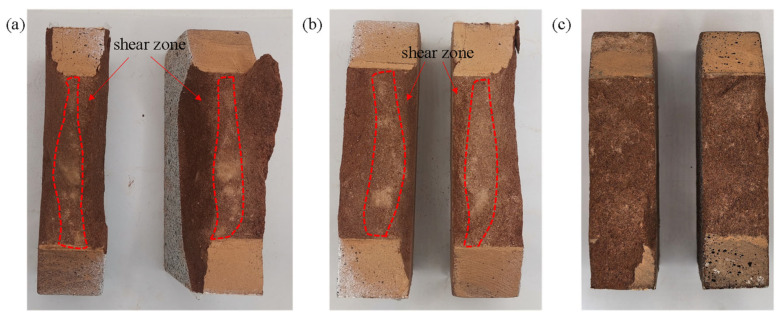
Fracture surfaces of red sandstone specimens: (**a**) RS-50-2, (**b**) RS-60-1, and (**c**) RS-70-1.

Due to differences in fracture mechanisms, specimens under different loading angles exhibit distinct fracture modes, which can be identified from the characteristics of fracture surface shown in Figure 16. Under loading angles of 50° and 60°, large amounts of fine rock powder spread over the fracture surface of the rock, serving as a typical feature of mode II fracture. However, under the loading angle of 70°, no whitish area is found on the fracture surface, and its fracture surface is rougher, which are typical characteristics of mode I fracture.

**Figure 17:**
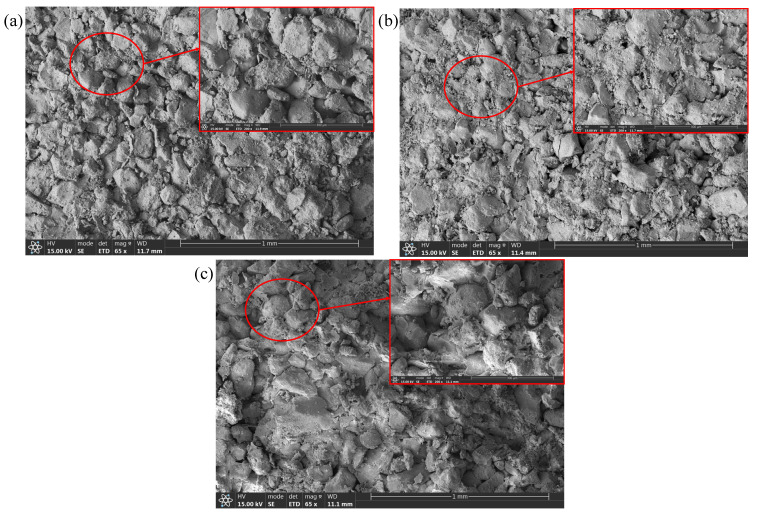
SEM images of fracture surfaces of specimens (**a**) RS-50-2, (**b**) RS-60-1, and (**c**) RS-70-1.

Additionally, SEM analysis can be employed to better understand the fracture mechanism of these fracture surfaces [27]. As shown in Figure 17, the microscopic fracture surfaces of the specimen under loading angles of 50° and 60° are relatively smooth, and many grains were crushed and ground flat. These typical characteristics demonstrate that the fractures under the loading angles 50° and 60° belong to shear fracture. However, the microscopic fracture surface of the specimen under a loading angle of 70° is rough and uneven, fewer grains were crushed, and fractures mainly propagated between grains. These typical characteristics demonstrate that the fracture under a loading angle of 70° belongs to tensile fracture.

## 4. Theoretical Analyses of Mode II Fracture Toughness

### 4.1. Stress Intensity Factor Analysis

**Figure 18:**
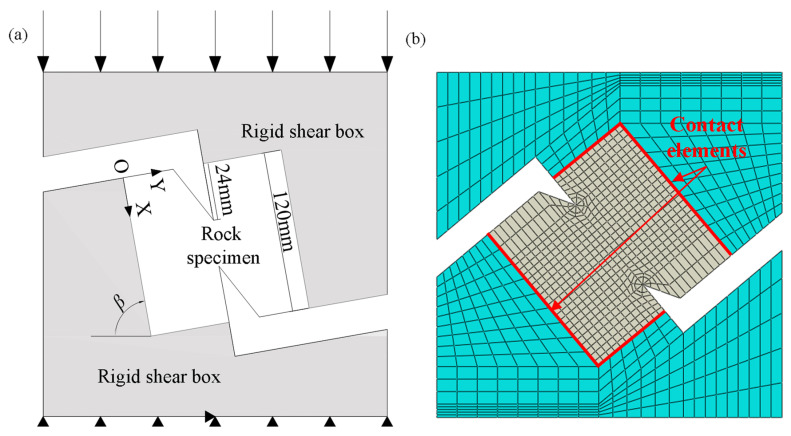
Finite element model of the shear box test. (**a**) Sketch of shear box test and (**b**) finite element meshes. (The arrows at the top represent the applied pressure, and the arrows at the bottom represent the applied constraint).

Finite element analysis was carried out to obtain the stress intensity factors and further analyze the crack initiation mechanism of red sandstone, and the shear box test was simplified to a plane stress problem. A square plane model with the size of 120 mm × 120 mm was established, and the mesh is shown in Figure 18. To improve the solution accuracy of the finite element stress intensity factor, a circular region with a radius of 5 mm is divided into meshes by using triangular singular cells radiating outward from the crack tip, while other regions are divided by using quadrilateral cells. At the crack tip, a crack with the extension direction pointing to the center of the specimen is set, the crack integral type is set as the stress intensity factor, and the intermediate node position parameter is set as 0.25. To investigate the actual contact between the rock specimen and the rigid loading device, the model of the loading device is established at the same time. Among them, the physical and mechanical parameters of the rock specimen are: $\rho_{r}=2800\;\mathrm{kg}/m^{3}$, $E_{r}=3.24\;\mathrm{GPa}$, and $\nu_{r}=0.279$. The material of the loading device is steel, and its physical and mechanical parameters are: $\rho_{s}=7800\;\mathrm{kg}/m^{3}$, $E_{s}=210\;\mathrm{GPa}$, and $\nu_{s}=0.25$. The contact relationship between the specimen and the loading device is simulated using contact elements. The normal contact behavior is hard contact, which allows the contact to be separated after tension [23,28,29]. The tangential contact behavior obeys Coulomb’s law with a friction coefficient of 0.3 [21]. To make the shear box uniformly loaded, the load is applied by a displacement control method, and a displacement load of 5 × 10^−5^ m is applied on the top surface of the shear box device. A vertical displacement constraint is applied on the bottom surface of the shear box device, and a horizontal displacement constraint is applied at the midpoint of the bottom surface. For all analyses, the width of the top and bottom surfaces of the shear box devices is 240 mm.

The mode I and mode II stress intensity factors, *K*_Ι_ and *K*_ΙΙ_, of the notch tip were solved by using the stress intensity factor algorithm integrated in Abaqus. The mode I and mode II stress intensity factors under the load condition 100 kN are listed in Table 3, in which the mode I stress intensity factors are all negative, indicating that the notch tip is in the state of compressive stress on the initial crack plane. Additionally, *K*_II_ gradually increases with the loading angle, while *K*_I_ remains essentially unchanged. *K*_II_ continues to increase with the loading angle, indicating that the loading angle has a more significant effect on *K*_ΙΙ_ and a relatively minor effect on *K*_Ι_ for the studied cases. Furthermore, to assess the effect of the friction coefficient on the analytical results, stress intensity factors for the specimen were further calculated under friction coefficients of 0.1 and 0.5, and the results are included in Table 3. When the contact friction coefficient ranges from 0.1 to 0.5, the maximum calculation errors for mode I and mode II stress intensity factors are only 1.07% and 0.66%, respectively. These results indicate that the influence of the friction coefficient on the calculation is negligible.

For a double-cracked square specimen with dimensions of *B* × *W* × *W* and crack length of *a* under shear box loading, it is necessary to introduce dimensionless shape factors *Y*_I_ and *Y*_II_ to characterize the effect of the specimen geometry on the stress intensity factors *K*_I_ and *K*_II_ [18]:

(1) $$\left\{\begin{array}{l} K_{I}=-\frac{P\cos\beta }{BW}\sqrt{\pi a}Y_{I}\left(\frac{a}{W},\beta \right)\\ K_{\mathrm{II}}=\frac{P\sin\beta }{BW}\sqrt{\pi a}Y_{\mathrm{II}}\left(\frac{a}{W},\beta \right) \end{array}\right.$$

where *B* is the thickness of the specimen, and *B* = 1, since the finite element model is analyzed using a planar model. *P* is the axial load applied by the loading device, and *β* is the loading angle of the specimen. The dimensionless shape factor is not only related to a/W but also affected by the loading angle *β*. According to the results of the finite element analysis of the stress intensity factor, the dimensionless stress intensity factor of mode I and mode II can be solved as:

(2) $$\left\{\begin{array}{l} Y_{I}\left(\frac{a}{W},\beta \right)=-\frac{BWK_{I}}{P\cos\beta \sqrt{\pi a}}\\ Y_{\mathrm{II}}\left(\frac{a}{W},\beta \right)=\frac{BWK_{II}}{P\sin\beta \sqrt{\pi a}} \end{array}\right.$$

where $Y_{I},\;Y_{\mathrm{II}}$ are mode I and mode II dimensionless shape factors.

**Figure 19:**
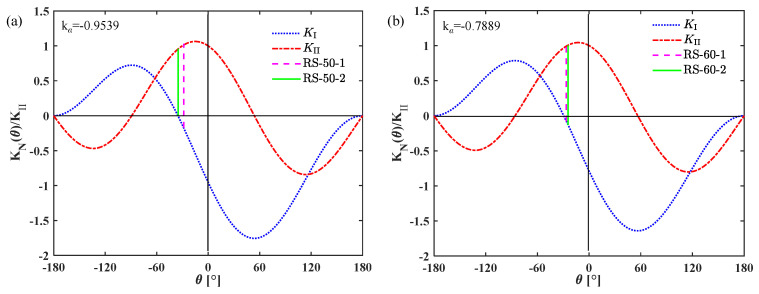
Variation in *K*_I_(*θ*) and *K*_II_(*θ*) and the crack initiation angles under different loading conditions. (**a**) *k*_α_ = −0.9539; (**b**) *k*_α_ = −0.7889.

The stress intensity factor for different angles in the polar coordinate system is expressed as [24]:

(3) $$\left\{\begin{array}{c} K_{I}(\theta )=K_{I}\cos^{3}\frac{\theta }{2}-3K_{I}\sin\frac{\theta }{2}\cos^{2}\frac{\theta }{2}\\ K_{I}(\theta )=K_{I}\sin\frac{\theta }{2}\cos^{2}\frac{\theta }{2}+K_{\mathrm{II}}\cos\frac{\theta }{2}\left(1-3\sin^{2}\frac{\theta }{2}\right) \end{array}\right.$$

where *θ* is the polar coordinate of the crack tip,

To facilitate the analysis, the loading ratio *k*_α_ is defined as *K*_I_/*K*_II_. In the shear box test, it is found that compression–shear fracture of the rock specimen occurs when the loading angle is 50° and 60°, so these two loading cases are further analyzed. According to Table 3, *k*_α_ = −0.9539 when the loading angle is 50°, and *k*_α_ = −0.7889 when the loading angle is 60°. According to Equation (3), the curves of *K*_I_(*θ*) and *K*_II_(*θ*) and the average crack initiation angles in these two loading cases are plotted in Figure 19. It demonstrates that the crack initiation plane is in a state of compression–shear stress, and the fracture that occurs is a compression–shear mixed-mode fracture.

### 4.2. Determination of Mode II Fracture Toughness

Rao et al. [17] found that in the shear box test with a loading angle between 65 and 75° and initial notch length ratio greater than 0.6, the cracks of double-notched specimens initiated from the tips of both notches and the experimental crack initiation angle was less than 5°, so they believed that cracks extended in the original crack plane. However, it was found in this study that the cracks in specimens with an initial notch length ratio of 0.4 were in the original crack plane at a loading angle of 70°, but the cracks were distributed in the center of the rock bridge and were characterized as tensile fracture. When the loading angle is 50° and 60°, the cracks initiate away from the original crack plane, and compressive-shear fracture occurs. Therefore, in this paper, we cannot follow their theory to solve the mode II fracture toughness of rocks but need to establish an analysis theory of mode II fracture toughness considering the effect of fracture angle.

When the fracture plane is subjected to compressive stress, the Mohr–Coulomb criterion is usually used to reflect the effect of compressive stress on the fracture. The Mohr–Coulomb criterion is expressed as:

(4) $$\tau_{f}=-\sigma_{n}f+c\;\left(\sigma_{n}\leq 0\right)$$

where c is the shear strength when the compressive stress is zero, often called cohesion, and f is the friction coefficient.

The Mohr–Coulomb criterion is not directly applicable to fracture analysis but needs to be converted to another form. This is achieved by multiplying both sides of Equation (4) by $\sqrt{2\pi r}$ simultaneously and taking the limiting value of *r,* which tends to zero. Therefore, when a compressive-shear crack initiates, the following relation can be established according to the Mohr–Coulomb criterion:

(5) $$\underset{r\to 0}{\lim}\left(\tau_{r\theta }\sqrt{2\pi r}=-\sigma_{\theta }\sqrt{2\pi r}f+c\sqrt{2\pi r}\right)$$

where *r* and *θ* are the polar coordinates of the crack tip, while $\sigma_{\theta }$ and $\tau_{r\theta }$ are the stress components in the polar coordinates around the crack tip.

By definition, $K_{I}\left(\theta \right)=\underset{r\to 0}{\lim}\sigma_{\theta }\sqrt{2\pi r}$, while $K_{\mathrm{II}}\left(\theta \right)=\underset{r\to 0}{\lim}\tau_{r\theta }\sqrt{2\pi r}$. When $K_{I}\left(\theta \right)=0$, the fracture mode is a pure mode II fracture controlled by the mode II fracture toughness *K*_IIC_. According to the above equation we can get $\underset{r\to 0}{\lim}c\sqrt{2\pi r}=K_{\mathrm{IIC}}$. Thus, the Mohr–Coulomb criterion can be expressed as an expression for the mode I stress intensity factor and pure mode II fracture toughness:

(6) $$K_{\mathrm{IIC}}^{\prime }\left(\theta \right)=K_{\mathrm{IIC}}-K_{I}\left(\theta \right)f$$

where $K_{\mathrm{IIC}}^{\prime }\left(\theta \right)$ is the fracture toughness considering the effect of normal compressive stress.

Prior to crack initiation, the shear stress intensity factor should satisfy the following conditions:

(7) $$K_{\mathrm{II}}(\theta )\leq K_{\mathrm{IIC}}-K_{I}(\theta )f$$

By moving $K_{I}(\theta )f$ to the left of Equation (7), a compressive-shear mixed-mode stress intensity factor after considering the Mohr–Coulomb criterion can be defined:

(8) $$K_{S}\left(\theta \right)=K_{\mathrm{II}}(\theta )+K_{I}(\theta )f$$

With this definition, *K*_S_ can be directly compared with the pure mode II fracture toughness *K*_IIC_ to identify the initiation of compressive-shear fracture. If the initiation angle of compression–shear fracture is defined as $\theta_{\mathrm{MC}}$, the compressive-shear mixed-mode fracture criterion can be expressed as:

(9) $$K_{S}\left(\theta_{\mathrm{MC}}\right)=K_{\mathrm{IIC}}$$

where $K_{\mathrm{IIC}}$ is the pure mode II fracture toughness. Therefore, in this paper, the pure mode II fracture toughness of the rock can be solved according to Equation (9).

In the solution method of pure mode II fracture toughness proposed by Rao et al. [17], the pure mode II fracture toughness for the shear box test was obtained by assuming that the crack initiates along the initial crack plane, and it is expressed as:

(10) $$K_{\mathrm{IIC}}=\frac{Q_{e}}{BW}\sqrt{\pi a}Y_{\mathrm{II}}\left(\frac{a}{W},\beta \right)$$

where the dimensionless shape factor is replaced by the mode II dimensionless shape factor calibrated in this paper, and $Q_{e}$ is an equivalent shear load considering the Mohr–Coulomb criterion:

(11) $$Q_{e}=P_{c}\left(\sin\beta -f\cos\beta \right)$$

where $P_{c}$ is the peak load.

To compare with their solution method of pure mode II fracture toughness, based on the assumption that the crack initiates along the initial plane ($\theta_{\mathrm{MC}}=0$), the proposed method can be converted into a mode II fracture toughness expression similar to Equation (10). When the crack initiates along the initial plane, the following equation is obtained by substituting $\theta_{\mathrm{MC}}=0$ into Equation (8):

(12) $$K_{S}\left(\theta_{\mathrm{MC}}\right)=K_{\mathrm{II}}+K_{I}f=K_{\mathrm{IIC}}$$

Substituting the expressions of $K_{I}$ and $K_{\mathrm{II}}$ in Equation (1) into the above equation yields:

(13) $$K_{\mathrm{IIC}}=\frac{P_{c}\sin\beta }{BW}\sqrt{\pi a}Y_{II}\left(\frac{a}{W},\beta \right)-\frac{P\cos\beta }{BW}\sqrt{\pi a}Y_{I}\left(\frac{a}{W},\beta \right)f$$

The above equation can be further simplified as:

(14) $$K_{\mathrm{IIC}}=\frac{Q_{e}^{\prime }}{BW}\sqrt{\pi a}Y_{\mathrm{II}}\left(\frac{a}{W},\beta \right)$$

where $Q_{e}^{\prime }$ is an equivalent shear load obtained by the proposed method:

(15) $$Q_{e}^{\prime }=P\left[\sin\beta -f\cos\beta \frac{Y_{I}\left(\frac{a}{W},\beta \right)}{Y_{\mathrm{II}}\left(\frac{a}{W},\beta \right)}\right]$$

A comparison of the equivalent shear loads presented in Equations (11) and (15) reveals that their solution method of mode II fracture toughness neglects the effect of the mode I dimensionless shape factor in addition to not considering the effect of the initiation angle.

A comparison of the pure mode II fracture toughness calculated by Equations (9), (10) and (14) is presented in Table 4. It shows that the pure mode II fracture toughness solved by their method is 0.16 and 0.65 $\mathrm{MPa}\cdot m^{1/2}$ under loading angles of 50° and 60°, respectively, and the differences are large, which does not reflect the irrelevance of the pure mode II fracture toughness to the confining pressure very well. The pure mode II fracture toughness solved by the proposed method is 1.60 and 1.98 $\mathrm{MPa}\cdot m^{1/2}$, respectively, which are closer to each other and can better reflect the irrelevance of the pure mode II fracture toughness to the confining pressure. According to the test results, the standard deviation of *K*_IIC_ is 0.27 $\mathrm{MPa}\cdot m^{1/2}$, and the 95% confidence interval of *K*_IIC_ is from 1.35 to 2.23 $\mathrm{MPa}\cdot m^{1/2}$. There is still a difference between the resulting fracture toughness from the two loading cases because the test results are affected by the tensile spalling on the specimen’s surface, which makes the area of the shear band vary with the specimen and loading condition. Additionally, their method underestimates the pure mode II fracture toughness. When their formulas are used to analyze the experimental results in this study, the computational errors of their formulas range from 59% to 92%, and the relative errors decrease with the increase in loading angle.

The difference between pure mode II fracture toughness solved by the above two methods is very large, and it is caused by the neglection of the mode I dimensionless shape factor and the initiation angle. Further analysis is carried out to investigate the main influence factor on the difference. The fracture toughness results obtained by Equation (14), which considers the influence of the mode I dimensionless shape factor, are also listed in Table 4. $Q_{e}^{\prime }$ is negative at a loading angle of 50°, which means that it is impossible to promote a compressive-shear fracture initiating on the original crack plane, so it is also impossible to solve for the positive *K*_IIC_ by $Q_{e}^{\prime }$. At a loading angle of 60°, the *K*_IIC_ calculated by Equation (14) is 0.23 $\mathrm{MPa}\cdot m^{1/2}$, and the relative error increases to 86%. Such an error is caused by the neglection of the initiation angle, so the crack initiation angle has a more significant influence on the determination of pure mode II fracture toughness than the mode I dimensionless shape factor. Overall, the influences of both the experimental crack initiation angle *θ* and the mode I dimensionless shape factor on the *K*_IIC_ are non-negligible.

## 5. Discussion

### 5.1. Comparison with the Punch-Through Shear Test

**Figure 20:**
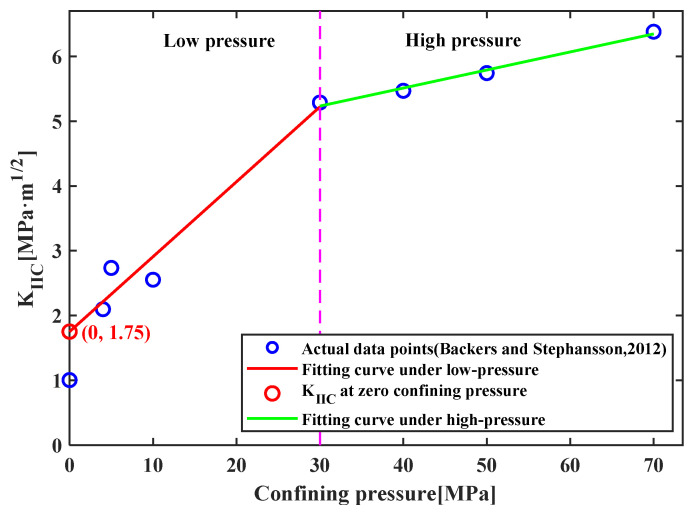
Mode II fracture toughness of red sandstone tested by the PTS test conducted by Backers and Stephansson [15]. (The pink dotted line represents the boundary between low-pressure and high-pressure areas).

To further illustrate the rationality of the proposed method, the results are compared with the results of the punch-through shear (PTS) test, which is a test method of mode II fracture toughness suggested by the International Society for Rock Mechanics. Backers and Stephansson [15] employed the PTS test to measure the mode II fracture toughness of red sandstone specimens under different confining pressures. They observed that the measured fracture toughness increased with the confining pressure, and the calculated *K*_IIC_ differs significantly under low- and high-confining-pressure conditions, which are divided by a confining pressure of 30 MPa, as shown in Figure 20. The data of the specimen under the condition of no confining pressure significantly deviates from the overall trend, so its fracture may not be caused by shear fracture but by tensile fracture. Additionally, it has become a recognized fact that rocks cannot undergo shear fracture without confining pressure [9,10]. The remaining *K*_IIC_ values are compressive-shear fracture toughness. Huang et al. [30] investigated the compression–shear fracture properties of sandstone materials exposed to confining pressure, finding that the fracture toughness of sandstone specimens satisfies linear correlation with the confining pressure. Thus, to obtain a pure mode II fracture toughness from their results, the trends of *K*_IIC_ under low- and high-confining-pressure conditions are fitted by two straight lines, respectively. The trend line of *K*_IIC_ has an intersection point with the vertical axis, which indicates a pure mode II fracture toughness of 1.75 $\mathrm{MPa}\cdot m^{1/2}$. The mode II fracture toughness of red sandstone determined by the proposed method is 1.60–1.98 $\mathrm{MPa}\cdot m^{1/2}$. Thus, the mode II fracture toughness values obtained by the two testing methods show good agreement. Compared with the PTS testing method, the shear box fracture test offers advantages in terms of simpler specimen preparation and easier observation of the crack propagation process, making it more suitable for investigations into shear fracture behavior. Therefore, the shear box fracture test is recommended for cases requiring observation of the crack propagation process.

In this paper, the fracture mechanisms of bilateral notched red sandstone specimens in shear box tests with different loading angles are investigated through laboratory tests, finite element simulations, and DIC analysis, and the determination method of mode II fracture toughness is modified by considering the experimental crack initiation angle and mode I dimensionless shape factor.

### 5.2. Limitations

While the integrated approach combining laboratory testing, finite element simulation, and DIC analysis in this study provides valuable insights into the fracture mechanisms and an improved method for determining mode II fracture toughness, several limitations inherent to the methodology and assumptions should be considered. These are summarized as follows:

(1) The heterogeneity of rock introduces two primary effects on the test results. Firstly, it contributes to the scatter observed in the measured mode II fracture toughness. Secondly, rock heterogeneity promotes local stress concentration, which weakens the overall strength of the rock and thereby reduces its mode II fracture toughness.
(2) The plane stress assumption may underestimate the fracture toughness. According to Akbardoost and Bidadi [31], mode II fracture toughness increases with specimen thickness when the thickness-to-size ratio is below 0.56. In this study, the ratio is 0.25, implying that the plane stress assumption may lead to an underestimation of the actual fracture toughness.
(3) The Mohr–Coulomb criterion relies on a linear strength envelope, which cannot adequately represent nonlinear material response under high confining pressure or strain-softening behavior. Since the crack tip is actually in a state of high confining pressure, the applicability of the Mohr–Coulomb criterion in such conditions therefore requires further verification.

## 6. Conclusions

This study investigated the fracture mechanisms of bilateral notched red sandstone under various loading angles in shear box tests, employing an integrated methodology of laboratory experiments, finite element simulations, and DIC analysis. A modified method for determining mode II fracture toughness was proposed by incorporating the experimentally observed crack initiation angle. The principal findings are summarized as follows:

(1) The peak load decreased with increasing loading angle. Fracture patterns transitioned from irregular and chaotic at low angles (30–40°) to regular shear fractures initiating from the notch tip with angles between −24.5° and −35° at intermediate angles (50–60°) and finally to tensile-dominated fracture along the original crack plane at 70°.
(2) DIC analysis elucidated the underlying deformation mechanisms at 50° and 60°, and combined compressive and shear deformation indicated a compressive-shear fracture mode, while the tensile strain field at 70° confirmed a tensile fracture mechanism.
(3) A theoretical framework based on the Mohr–Coulomb criterion was developed to calculate pure mode II fracture toughness for compressive-shear fractures. Applying this method yielded values of 1.60 $\mathrm{MPa}\cdot m^{1/2}$ and 1.98 $\mathrm{MPa}\cdot m^{1/2}$ at 50° and 60°, respectively. The traditional method, neglecting the initiation angle and mode I dimensionless shape factor, introduced significant errors (59–92%), with the initiation angle being the most critical parameter.

The core methodological advancement is the integration of measured crack initiation angles into the toughness calculation, moving beyond purely theoretical or empirical assumptions. The primary engineering value of this study lies in providing a more accurate and mechanically consistent method to assess the in situ shear strength of rock masses, which is crucial for stability analysis in slopes, underground excavations, and dam foundations.

This study has certain limitations. The small specimen number per group, though a necessary compromise for material consistency, affects statistical robustness. The plane stress assumption and the use of the linear Mohr–Coulomb criterion at the high-confining-pressure crack tip region require further validation. Future research should focus on: (1) conducting tests with a larger number of specimens and different rock types to enhance statistical significance and generality; (2) performing 3D analyses or tests with varying thicknesses to evaluate the plane stress/strain assumption; and (3) developing or applying nonlinear failure criteria that better capture post-peak and high-confining-pressure behavior for more accurate fracture predictions.

## Figures and Tables

**Table 1:** Information on red sandstone specimens.

Specimen Number	Loading Angle [°]	Length of Edges Without Notch [mm]	Length of Edges with Notch [mm]	Thickness [mm]	Depth of Notch A [mm]	Depth of Notch B [mm]	Mass [g]	Density [g/cm^3^]
RS-30-1	30	119.99	120.07	30.34	24.12	24.10	924.7	2.166
RS-30-2	30	119.64	119.83	30.11	23.95	24.11	926.2	2.197
RS-40-1	40	120.02	120.03	30.27	23.97	23.98	917.9	2.155
RS-40-2	40	119.98	119.92	30.23	24.22	23.92	915.1	2.154
RS-50-1	50	120.04	119.97	30.29	24.03	24.07	922.6	2.165
RS-50-2	50	119.86	119.79	30.11	23.85	24.00	884.1	2.093
RS-60-1	60	119.90	119.89	30.13	24.17	24.26	895.4	2.117
RS-60-2	60	119.82	119.74	30.20	24.20	24.09	912.5	2.157
RS-70-1	70	119.60	119.80	30.32	23.92	23.63	905.4	2.133
RS-70-2	70	120.12	120.33	30.39	24.26	24.15	925.5	2.157
Minimum		119.6	119.74	30.11	23.85	23.63	884.1	2.093
Maximum		120.12	120.33	30.39	24.26	24.26	926.2	2.197
Mean		119.90	119.94	30.24	24.07	24.03	912.94	2.15
Standard deviation		0.162	0.166	0.095	0.136	0.161	13.382	0.027

**Table 2:** Crack initiation angles of red sandstone specimens.

Specimen Number	Overall Fracture Pattern	Notch Position	Order of Fracture	Initiation Angles in the Front [°]	Initiation Angles in the Back [°]	Consistent?	Average
RS-30-1	chaotic	Notch A	2nd	−38	−21	Almost	−29.5
		Notch B	1st	17	17	Very	17
RS-30-2	chaotic	Notch A	2nd	\	29	Not	
		Notch B	1st	−78	−44	Not	
RS-40-1	chaotic	Notch A	1st	21	34	Almost	27.5
		Notch B	2nd	\	−19	Not	
RS-40-2	chaotic	Notch A	2nd	\	\	Not	
		Notch B	1st	23	24	Very	23.5
RS-50-1	regular	Notch A	2nd	−31	−31	Very	−31
		Notch B	1st	−26	−26	Very	−26
RS-50-2	regular	Notch A	1st	−38	−32	Very	−35
		Notch B	2nd	\	\	Not	
RS-60-1	regular	Notch A	2nd	\	10	Not	
		Notch B	1st	−25	−28	Very	−26.5
RS-60-2	regular	Notch A	2nd	11	\	Not	
		Notch B	1st	−23	−26	Very	−24.5
RS-70-1	regular	Notch A	2nd	7	13	Very	10
		Notch B	1st	0	−5	Very	−2.5
RS-70-2	regular	Notch A	2nd	−8	−10	Very	−9
		Notch B	1st	0	9	Very	4.5

Note: “\” denotes no fracture initiating from this notch. The judgment standard for “no fracture initiation” is based on visual observation of the final fracture morphology on the specimen’s surface.

**Table 3:** Mode I and mode II stress intensity factors.

Loading Angle [°]	Friction Coefficient of Contact Behavior	*K*_I_ $[\mathrm{MPa}\cdot m^{1/2}]$	*K*_II_ $[\mathrm{MPa}\cdot m^{1/2}]$	*K*_I_/*K*_II_	Error Due to the Influence of Friction Coefficient	
					*K*_I_	*K*_II_
30	0.3	−0.2264	0.1387	−1.6323	\	\
40	0.3	−0.2163	0.1803	−1.1997	\	\
50	0.3	−0.2126	0.2229	−0.9539	\	\
60	0.3	−0.2177	0.2760	−0.7889	\	\
70	0.3	−0.2209	0.3492	−0.6326	\	\
30	0.1	−0.2432	0.1496	−1.6258	1.07%	0.66%
40	0.1	−0.2012	0.1684	−1.1942	1.03%	0.57%
50	0.1	−0.1668	0.1757	−0.9496	1.01%	0.56%
60	0.1	−0.1393	0.1774	−0.7852	0.90%	0.44%
70	0.1	−0.1081	0.1716	−0.6297	0.85%	0.41%
30	0.5	−0.2481	0.1514	−1.6385	−0.92%	−0.56%
40	0.5	−0.2051	0.1703	−1.2046	−0.91%	−0.50%
50	0.5	−0.1700	0.1775	−0.9577	−0.88%	−0.48%
60	0.5	−0.1416	0.1788	−0.7920	−0.76%	−0.37%
70	0.5	−0.1098	0.1729	−0.6349	−0.72%	−0.34%

**Table 4:** Comparison of three methods for solving fracture toughness.

Specimen Number	Actual Cracking Angle [°]	K_IIC_ Calculated by the Proposed Method $[\mathrm{MPa}\cdot m^{1/2}]$		K_IIC_ Calculated by Equation (10) $[\mathrm{MPa}\cdot m^{1/2}]$			K_IIC_ Calculated by Equation (14) $[\mathrm{MPa}\cdot m^{1/2}]$		
		Each	Average	Each	Average	Relative Error	Each	Average	Relative Error
RS-50-1	−26	1.84	1.98	0.17	0.16	92%	\	\	\
RS-50-2	−35	2.12		0.15			\		
RS-60-1	−26.5	1.75	1.60	0.69	0.65	59%	0.24	0.23	86%
RS-60-2	−24.5	1.45		0.60			0.21		

## Data Availability

The original contributions presented in this study are included in the article. Further inquiries can be directed to the corresponding author.

## Nomenclature

$K_{\mathrm{IC}},\;K_{\mathrm{IIC}}$	Mode I and mode II fracture toughness
$\rho_{r},\;E_{r},\nu_{r}$	Density, elastic modulus, and Poisson’s ratio of rock
$\rho_{s},\;E_{s},\;\nu_{s}$	Density, elastic modulus, and Poisson’s ratio of steel
a, W, B	Notch depth, side length, and thickness of specimen
β	Loading angle of specimen
2a/W	Initial notch length ratio
$P_{n},\;P_{s},\;P_{c}$	Normal force, tangential force, and peak load
θ	Angle of potential fracture plane
$u_{1},\;v_{1}$	Normal displacement perpendicular and tangential displacement parallel to fracture
$\varepsilon_{\mathrm{xy}},\;\varepsilon_{\mathrm{yy}},\;\varepsilon_{\mathrm{xx}}$	Strain components in cartesian coordinate system
$K_{I},\;K_{\mathrm{II}}$	Mode I and mode II stress intensity factor on the original crack plane
$Y_{I},\;Y_{\mathrm{II}}$	Mode I and mode II dimensionless shape factor
$\sigma_{r},\;\sigma_{\theta },\;\tau_{r\theta }$	Stress components in polar coordinate system around crack tip
$K_{I}\left(\theta \right),\;K_{\mathrm{II}}\left(\theta \right)$	Mode I and mode II stress intensity factors on *θ* plane
$k_{\alpha }$	Loading ratio, $K_{I}/K_{\mathrm{II}}$
$\sigma_{\mathrm{xx}},\;\sigma_{\mathrm{yy}},\;\tau_{\mathrm{xy}}$	Stress components in cartesian coordinate system
$\sigma_{n}$	Normal stress on *θ* plane
c	Shear strength when the compressive stress is zero
f	Friction coefficient
ϕ	Fracture angle under uniaxial compression
$K_{\mathrm{IIC}}^{\prime }\left(\theta \right)$	Fracture toughness considering the effect of normal compressive stress
$\theta_{\mathrm{MC}}$	Initiation angle of compressive-shear fracture
$Q_{e}$	Equivalent shear load
$Q_{e}^{\prime }$	Modified equivalent shear load
